# Screening and identification of differentially expressed transcripts in circulating cells of prostate cancer patients using suppression subtractive hybridization

**DOI:** 10.1186/1476-4598-4-30

**Published:** 2005-08-08

**Authors:** Xin Li, Carson Wong, Ralph Mysel, Gennady Slobodov, Adam Metwalli, Jarrett Kruska, C Scott Manatt, Daniel J Culkin, Bradley P Kropp, Hsueh-Kung Lin

**Affiliations:** 1 Department of Urology, University of Oklahoma Health Sciences Center, Oklahoma City, OK 73104, USA

**Keywords:** circulating tumor cells, suppression subtractive hybridization, prostate cancer

## Abstract

**Background:**

Tumor metastasis and changes in host immunosurveillance are important components in cancer development. Tumor cell invasion into the bloodstream is an essential step for systemic metastasis. Currently, the detection of tumor cells in the circulation is mainly dependent upon the utilization of known epithelial cell markers. However, expression of these molecules is not limited to cancer patients; healthy people also have a small number of epithelial cells in their circulation. Utilizing these markers to detect circulating tumor cells (CTCs) cannot adequately explain the mechanisms of tumor cell survival or their development of metastatic potential in peripheral blood. The immune system can also evolve along with the cancer, actually promoting or selecting the outgrowth of tumor variants. Unfortunately, both metastasis and immunosurveillance remain mysterious and are debatable because we have yet to define the molecules that participate in these processes. We are interested in identifying the existence of expressed genes, or mRNA species, that are specifically associated with circulating cells of cancer-bearing patients using prostate cancer (PCa) as a model.

**Results:**

We established two comprehensive subtracted cDNA libraries using a molecular technique called *suppression subtractive hybridization*. This technique selectively amplifies transcripts that are specifically expressed in circulating cells of either PCa patients or healthy men. Following sequencing reaction, we showed that 17 out of 23 (73.9%) sequenced clones did not match any mRNAs in the GenBank database. This result suggests that genes associated with alterations in circulating cells of cancer-bearing patients are largely unknown. Semi-quantitative RT-PCR confirmed that two genes are up-regulated in circulating cells of PCa patients, whereas another two genes are down-regulated in the same patients.

**Conclusion:**

The comprehensive gene expression analysis is capable of identifying differentially expressed genes in circulating cells of healthy men and PCa patients. We did not attempt to enrich specific cell types in this study because phenotypes of CTCs and subsets of leukocytes participating in immunosurveillance remain largely unknown. Continuous studies of these differentially expressed genes will eventually lead us to understand the mechanisms involved in tumor metastasis and immune modulation during cancer development.

## Background

Metastasis is a sequential, multi-step process in which tumor cells detach from the primary tumor, migrate through the basement membrane and extracellular matrix, and invade the lymphatic and/or blood systems [1]. This is followed by the establishment of secondary tumors at distant sites. It has been suggested that tumor cell invasion into the bloodstream can occur earlier than the time of primary diagnosis [2]. The ability to detect occult tumor cells with metastatic potential could have a substantial clinical impact on the management of cancer patients. Most, if not all, markers developed to detect occult tumor cells of epithelium origin in peripheral blood have been based on the concept that circulating tumor cells (CTCs) continue to express epithelial cell markers [3]. Based on this concept, several epithelial cell markers have been evaluated for detecting disseminated tumor cells in the blood circulation. Frequently used molecules include cytokeratins (CKs) 7, 19, and 20 [4-6], carcinoembryonic antigen (CEA) [7,8], epidermal growth factor receptor [9] including HER-2/neu [10], mucin-1 [11], β-subunit of human chorionic gonadotropin (β-hCG) [12], and α-fetoprotein [13]. In prostate cancer (PCa) patients, the expression of prostate specific antigen (PSA) [14-16], prostate-specific membrane antigen (PSMA) [17,18], and human glandular kallikrein 2 (hK2) [19] along with other epithelial cell markers has been investigated individually or in combination [20] for their ability to detect CTCs in patients with localized and metastatic PCa. This detection strategy involves the amplification of target mRNAs species by reverse transcriptase-polymerase chain reaction (RT-PCR) [21-23]. However, the use of these markers to detect CTCs fails to explain mechanisms that regulate tumor cell survival in the circulation and the development of their metastatic capability.

Recent reports have also emphasized that the immune system actively participates in cancer formation and development. Although this concept of immune response was formulated more than half a century ago [24], the existence of cancer "immunosurveillance" is still largely unknown and debatable because we know very little about the molecules participating in this event. If cancer "immunoediting" is present under the concept of cancer immunosurveillance, we hypothesized that genes expressed in immune cells participating in this event are significantly different from their counterparts in healthy persons.

We also hypothesized that both CTCs and immune cells need to evolve through their gene expression at stages of cancer formation and progression. The identified mRNA species associated with circulating cells of cancer-bearing patients will serve as independent markers for future tumor staging and help us understand metastasis and immunosurveillance. In this study, using PCa as a model, we applied the suppression suppressive hybridization (SSH) technique [25] to establish two libraries consisting of mRNA species that are either present or absent in circulating cells of PCa patients. We sequenced a small number of clones present in these libraries, and identified that the majority, 17 out of 23 (73.9%), of the sequenced clones did not match previously identified mRNA species. From the sequenced clones, we confirmed that four genes are differentially expressed in circulating cells of healthy men and PCa patients using semi-quantitative RT-PCR. Two mRNA species were identified to be significantly elevated in PCa patients, and two mRNA species were identified to be significantly suppressed in PCa patients.

## Results and Discussion

Current protocols for detecting CTCs mainly utilize known epithelial cell markers or other tissue-specific molecules [26]. However, the presence of these markers in CTCs does not correlate with their survival in the circulation and their metastatic capability. Furthermore, molecules that participate in the process of "immunosurveillance" remain poorly understood. In this report, we used a PCR-based, genome-wide gene expression analysis named SSH to establish comprehensive, subtracted cDNA libraries to catalogue mRNA species either present or absent in circulating cells of PCa patients.

**Figure 1:**
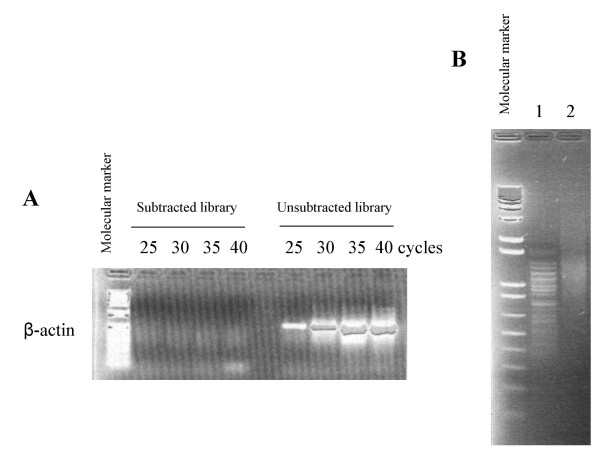
**Evaluation of subtraction efficiency and the presence of potential differentially expressed genes in the subtracted libraries. **To determine the subtraction efficiency, β-actin was PCR amplified using a primer set located within the very 3'-end of *Rsa *I digested β-actin fragment following the second round of hybridization. PCR products were electrophoresed on an agarose gel. No β-actin product was detected after 40 cycles of PCR amplification in a subtracted library, whereas β-actin was detected after 25 cycles of amplification in corresponding un-subtracted library (A). To amplify differentially expressed genes in circulating cells of healthy men and PCa patients, two rounds of PCR amplification was performed following hybridization steps described in *Materials and Methods*. To demonstrate the presence of potential differentially expressed genes in the subtracted libraries, the final PCR products were analyzed on a 1.5% agarose gel followed by ethidium bromide staining. We detected a series of distinct bands ranging from 300 to 1,000 bp. These DNA fragments represented genes that are either present (B, lane 1) or absent (B, lane 2) in circulating cells of PCa patients.

The SSH libraries were constructed from two age- and race-matched, pooled sample populations, healthy men and PCa patients. Each pooled sample consisted of 25 individual double-stranded cDNA libraries derived from circulating cell poly(A)^+ ^RNA of 25 men. We used a PCR-based method to evaluate the hybridization efficiency. After two rounds of hybridization, β-actin was amplified from a subtracted population using a pair of gene-specific primers located within the very 3' end of *Rsa *I digested β-actin. No DNA product was detectable after 40 cycles of amplification (Figure 1A), whereas the corresponding un-subtracted library showed the presence of abundant β-actin. This result demonstrated that β-actin, and possibly the majority of commonly expressed genes between the two sample populations, formed heterohybrids, and could not be amplified using the suppression PCR technique [25]. To demonstrate that the subtracted cDNA libraries contain potential differently expressed mRNA species, the PCR products were electrophoresed on an agarose gel following the second round of PCR. A series of DNA fragments, ranging from 300 to 1,000 bp in size, representing mRNA species specifically expressed in PCa patients were detected. The various PCR products represent the *Rsa *I digested cDNA fragments are shown in Figure 1B, lane 1. These results indicated that there are differentially expressed genes present only in the circulating cells of PCa patients but not healthy men. PCR products representing genes expressed only in the circulating cells of healthy men are shown in Figure 1B, lane 2.

To reveal the identities of the cDNA clones, the second round PCR products from both subtracted libraries were subcloned into the pCRII TA cloning vector. A total of 23 clones from both subtracted libraries were randomly selected for sequencing using M13 reverse primer. Identities of these clones are listed in Table 1. A majority of the sequenced clones, 17 out of 23 (73.9%), matched to genomic DNA fragments in the GenBank database, but not previously identified mRNA species. These results might reflect that some of these clones were amplified from rare CTCs in PCa patients and mRNA species reflecting these cells' biology or pathology have not been identified using traditional cDNA construction and sequencing. In addition, it is possible that genes identified from both subtracted libraries may represent previously un-identified molecules participating in tumor-immune system interactions [27-30].

**Table 1:** Identities of selected cDNA clones present in subtracted libraries

Clone I.D.	GenBank Accession No.	Gene Description
PCa-001*	AC026205	Homo sapiens chromosome 3 clone RP11-61I9 map 3p, complete sequence
PCa-002	AC019106	Homo sapiens BAC clone RP11-479L11 from 2, complete sequence
PCa-004	AY341247.1	Homo sapiens integral membrane protein 2B (ITM2B) gene, complete cds
PCa-005	AC104771.4	Homo sapiens BCA clone RP11-1E1 from 4, complete sequence
PCa-006	AC132068	Homo sapiens chromosome 16 clone CTD-2326c4, complete sequence
PCa-007	AK091994	Home sapiens cDNA FLJ34675 fis, clone Liver2001608
PCa-008	AC092910.9	Homo sapiens 3 BAC Rp11-767L7 (Roswell Park Cancer institute human BCA Library)
PCa-009	AC004690.2	Homo sapiens PAC clone RP-630c24 from 7, complete sequence
PCa-010	AC097461	Homo sapiens bCA clone RP11-6P6 from 2, complete sequence
PCa-011	BC047553	Homo sapiens calmodulin 2 mRNA (phosphorylase kinase δ)
PCa-012	AL031274	Homo sapiens chromosome 1q24 (clone RP4-798A17) contains the 3' part of the FMO1 gene and the FMO4 gene
PCa-013	AC010369	Homo sapiens chromosome 5 (clone CTC-2048F20)
PCa-014	NG_002397	Homo sapiens major histocompatibility complex, class I, BC (HLA-BC)
PCa-015	BC016320	Homo sapiens cathepsin D (Lysosomal aspartyl protease) mRNA
PCa-016	AC021701	Homo sapiens chromosome 18 (clone RP11-704G7)
Nrml-001**	AC004914.1	Homo sapiens PCA clone RP5-88608 from 7, complete sequence
Nrml-002	AK095899.1	Homo sapiens cDNA FLJ38580 fis, clone HCHON2008582, highly similar to ferritin heavy chain
Nrml-003	AC006083	Homo sapiens chromosome 17, clone hRPK.1053_B_8, complete sequence
Nrml-004	AL109759.4	Human chromosome 14 DNA sequence BAC R-898B23 of library RPCI-11 from chromosome 14 of Homo sapiens (Human), complete sequence
Nrml-005	AK026823.1	Homo sapiens cDNA: FLJ23170 fis, clone LNG09984
Nrml-006	AC019335.5	Homo sapiens chromosome 8, clone RP11-453N18, complete sequence
Nrml-007	AL162252.17	Human DNA sequence from clone RP11-55J24 on chromosome 9, complete sequence
Nrml-008	AC016644.9	Homo sapiens chromosome 5 clone RP11-52M14, complete sequence

* PCa clones were selected from the subtracted cDNA library that represents mRNA species only present in circulating cells of PCa patients.

** Nrml clones were selected from the subtracted cDNA library that represents mRNA species absent in circulating cells of PCa patients but present in their counterparts in cancer-free healthy men.

**Figure 2:**
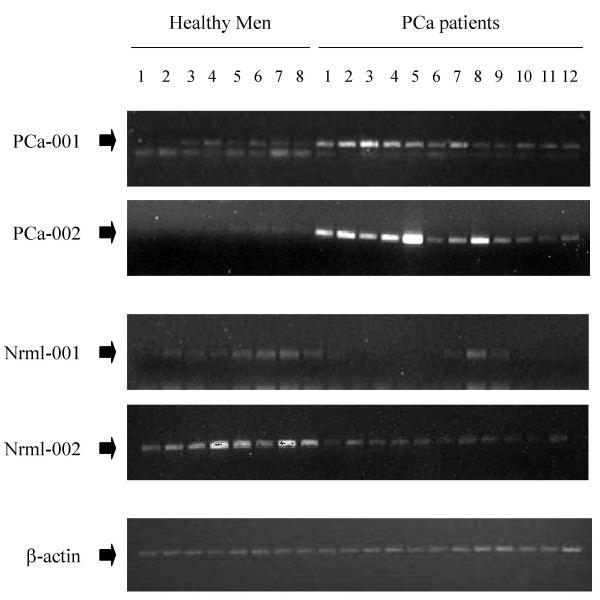
**Confirmation of differential gene expression in circulating cells of healthy men and PCa patients using semi-quantitative RT-PCR. **RT-PCR was performed on individual samples from 8 healthy controls and 12 PCa patients to confirm the SSH results. After sequencing reaction to reveal the identities of a total of 23 clones present in the subtracted libraries, PCR primers were designed (Table 2). β-actin was also amplified from the same samples using a β-actin primer set (BD Bioscinces Clontech) to serve as an internal control for standardizing the quantity of the RNA applied in each reaction. After PCR amplification, aliquots (10 μl) of these PCR products were electrophoresed into 2% agarose gels followed by ethidium bromide staining.

**Figure 3:**
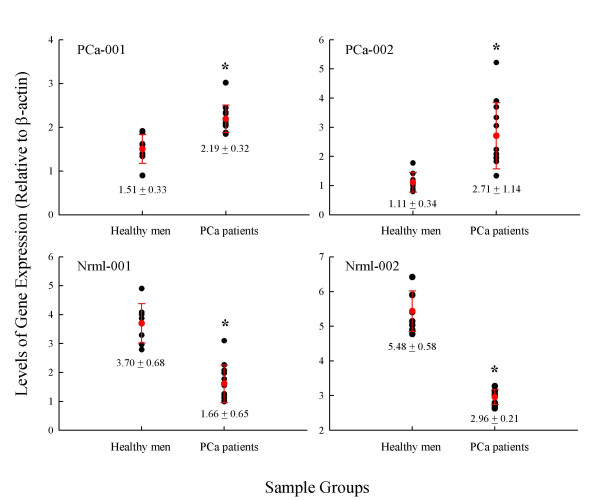
**Relative levels of target genes expression in peripheral blood circulating cells of healthy men and PCa patients. **Images obtained from Figure 2 were captured and analyzed using the Quantity One^® ^software. For each target gene, levels of gene expression were normalized to the level of β-actin expression for each individual sample. * indicates statistical significance between healthy men and PCa patients at *p *< 0.05.

Since a portion of the subtracted cDNAs may be false positive clones [25], we needed to confirm the identified clones as truly differentially expressed genes in our two sample populations. We used semi-quantitative RT-PCR to confirm that the cloned cDNAs are associated with peripheral blood circulating cells of cancer-bearing patients. Samples were collected from 12 PCa patients and 8 age- and race-matched healthy men for this analysis. RT-PCR was performed on individual samples. A total of 20 samples, 8 healthy men and 12 PCa patients, total RNA was analyzed for selected genes. Four target genes were selected to be confirmed by RT-PCR. Two genes, PCa-001 and PCa-002, were selected from the library that consists of mRNA species only present in circulating cells of PCa patients. PCR primers were designed according to the sequencing results (Table 2) and the designed primers were subjected to a BLAST search to ensure that these sequences do not match any identified mRNA with high homology. As expected, PCa-001 and PCa-002 were expressed at significantly higher levels in circulating cells obtained from PCa patients than in healthy men (Figure 2). The detection of low levels of PCa-001 and PCa-002 may due to high sensitivity of RT-PCR-based detection. Martin *et al*. also reported low levels of Mdm-2 and Gro-alpha expression in peripheral blood mononuclear cells of healthy samples using RT-PCR, whereas array-based analysis did not show a detectible signal for these two genes [31]. It is also possible that a very small number of epithelial cells can be present in the peripheral blood of healthy men [32] and PCa-001 and PCa-002 are epithelial cells markers. Another two genes, Nrml-001 and Nrml-002, were selected as absent in PCa patients' circulating cells. These two genes were expressed, as expected, at higher levels in healthy men than in PCa patients (Figure 2). β-actin has been shown to be constantly expressed in leukocytes of different individuals [33]. We also demonstrated that level β-actin expression is similar in all our samples (Figure 2). The relative levels of PCa-001, PCa-002, Nrml-001, and Nrml-002 expression were then normalized to β-actin and illustrated in Figure 3. Levels of these genes' expression were statistically different between healthy men and PCa patients.

**Table 2:** PCR primers and conditions for detecting levels of mRNA expression in circulating cells of healthy men and patients with PCa

Clone I.D.	GenBank Accession No.	PCR primers	cycles
PCa-001	AC132068	5'-AGG AAT AAG TCA CAC CGT GGA-3' 5'-ACC TGT TGG GAC TAG ACG CAT-3'	20
	nested	5'-TGG TCT GTA ACC CTT AGG AGA-3' 5'-TCT GCC CTT TGA GTC CAA GT-3'	25
Pca-002	AC019106	5'-AGG TCA GCA GAG ATG TCT GT-3' 5'-TAG TCC CCG AGA AAG AAT TA-3'	32
Nrml-001	AC004690	5'-TGA GCA GTT TCT TCA GCC TCA-3' 5'-TGA TAA GTC CAA CCC AAA GGC T-3'	20
	nested	5'-TAT CTG GGT GAC ACT GGG AAA-3' 5'-AGA GAC CAG CGT AAT ATC CCT-3'	30
Nrml-002	AK095899	5'-AGG TAA AGG AAA CCC CAA CAT GCA-3' 5'-AAC CAA CGA GGT GGC CGA ATC TT-3'	35

In order to ensure that the PCR products were not resulted from genomic DNA contamination, we performed two pre-cautionary procedures. First, all total RNA samples were subjected to RNase-free, DNase I digestion to remove residual genomic DNA. Second, we noticed that, in reactions with reverse transcription, β-actin signal was detected after 26 cycles of PCR amplification (Figure 2), whereas β-actin signal was absent in reactions without reverse transcription even after 35 cycles of amplification using amplimers within the same exon (data not shown). However, before we can confirm our statement, full-length cDNAs corresponding to our identified sequences need to be cloned.

The genome-wide screening and identification of mRNA species associated with circulating cells of tumor-bearing patients has been described for various cancers. Twine *et al*. reported the use of the microarray approach to differentiate gene expression patterns of mononuclear cells in patients with advanced renal cell carcinoma [34]. However, due to the low number of tumor cells present in the circulation compared to the large number of normal leukocytes, hybridization array (cDNA array and oligonucleotide array) may not be an ideal tool for identifying rare molecular events occurring in small number of CTCs. To overcome this problem, Smirnov *et al*. used magnetic separation of epithelial cell adhesion molecule (EpCAM) expressing cells from peripheral blood circulating cells and compared gene expression patterns between EpCAM-enriched and EpCAM-depleted cells using microarray analysis in cancer-bearing patients [35]. Another comprehensive gene expression method, named *mRNA differential display*, has been conducted to compare genes that are expressed in peripheral blood mononuclear cells of tumor-free individuals with those from lung, breast, and colon cancer patients [36]. This study found a total of 21 mRNA species expressed in tumor patients' blood samples but not in samples from tumor free volunteers. In addition, Martin *et al*. reported the use of the differential display to first identify transcripts differentially expressed in breast cancer cells and normal breast epithelial cells followed by an array analysis of these transcripts in the circulating cells of breast cancer patients [31]. Results from these experiments demonstrate that the detection of disseminated cancer cells in peripheral blood is attainable.

It has been suggested that selective enrichment of the tumor cell population from both bone marrow and blood before analysis can increase the sensitivity for detecting CTCs [37-40]. Various cell separation techniques have been devised to enrich the CTC population from whole blood. However, these methods may also introduce artifacts into the sample preparation steps. For example, the addition of anticoagulants to blood samples affect leukocyte gene expression *ex vivo *[41-43]. Efforts were made to avoid RNA degradation and alteration in gene expression during *in vitro *processing of blood cells and to avoid under- and over-estimation of *in vivo *mRNA expression. We used direct isolation of poly(A)^+ ^RNA and total RNA from whole blood circumventing a prior cell separation step. Our intention was also to prevent the loss of rare, un-identified target cells from blood samples during enrichment procedures since we do not know much about tumor cells' genotypes/phenotypes or any type(s) of immune cells' participation in cancer development.

Although it has been suggested that cancers are composed of a heterogeneous collection of cells with different degrees of tumor marker expressions [44,45], CTCs of all types might need to develop a "common" mechanism(s) to survive in the circulation and acquire metastatic capability. We speculate that universal "tumor-specific" markers can be identified in occult tumor cells from different cancers. Moreover, we expect to identify "tissue-specific" molecules in CTCs if tumor cells continue to express their tissue-specific markers. The identification of tissue-specific markers will help to identify the origins of CTCs. Emerging evidence also demonstrates that detection of tumor cells disseminated in peripheral blood can provide clinically important data for tumor staging, prognostication, and identification of surrogate markers for early assessment of the effectiveness of adjuvant therapy. Furthermore, by comparing gene expression profiling of all circulating cells, we expect to identify genes that might play a role in "immunosurveillance". Our future objectives include the identification of cell types that expressed differentially regulated mRNA species. We also intend to study the functional activities of these molecules in circulating cells during cancer development and establish an association between these genes' expression and cancer stages.

## Conclusion

Using the PCR-based SSH technique, we established two comprehensive subtracted cDNA libraries consisting of potentially differentially regulated genes in circulating cells of PCa patients. We further confirmed that both elevated and suppressed transcripts can be detected in circulating cells of PCa patients. This is an initial attempt to perform genome-wide gene expression analysis in peripheral blood circulating cells and demonstrate the presence of previously un-identified mRNA species in circulating cells of cancer-bearing patients. This is the first step toward understanding tumor metastasis and tumor-induced immune reactions in the development of cancer. We will continue to investigate these molecules' physiological/pathological function and their use in cancer detection.

## Methods

### Patient selection

PCa patients were enrolled at the time of diagnosis of elevated PSA and positive biopsy. Healthy men's samples were collected from volunteers with similar age and race distribution without evidence of diseases or use of any medications. Attending physicians provided all participants with informed consent forms for collecting samples used in this study. Sample collection was also HIPAA compliant. Blood was drawn before scheduled surgery from PCa patients. There was no evidence of systemic metastases for all PCa patients when the primary tumor was resected through surgical prostatectomy. For initial construction of SSH libraries, we collected 50 samples, 25 healthy men and 25 patients with PCa. We collected an additional 20 blood samples, 8 healthy men and 12 patients with PCa, for RT-PCR analysis.

### Blood collection and RNA isolation

For SSH, whole blood (5 ml) drawn from each individual was immediately mixed with 10x volume of RNA stabilization reagents for blood/bone marrow (Roche). The cells were then lysed. Poly(A)^+ ^RNA was immediately isolated by a two-step procedure through magnetic separation using the mRNA isolation kit for blood/bone marrow (Roche). The poly(A)^+ ^enriched samples were finally eluted from magnetic beads with H_2_O. Purified poly(A)^+ ^RNA was quantitated spectrophotometrically and stored in liquid nitrogen until use.

For RT-PCR, blood (2.5 ml) from each individual was colleted into a PAXgene™ Blood RNA tube (QIAGEN) following the manufacturer's protocol. Whole blood was thoroughly mixed with PAXgene stabilization reagent and stored at room temperature for 6 hours prior to RNA extraction. Total RNA was then extracted using the PAXgene™ Blood RNA kit according to the manufacturer's directions (QIAGEN). As the resulting RNA was usually contaminated with genomic DNA [46], total RNA samples absorbed to the PAXgene™ Blood RNA System spin column were incubated with DNase I (QIAGEN) at 25°C for 20 min to remove genomic DNA. Total RNA was eluted, quantitated, and stored in liquid nitrogen.

### Suppression subtractive hybridization (SSH) procedures

SSH was performed according to procedures described by Diatchenko *et al*. [25]. All reagents are now commercially available from BD Biosciences Clontech. Briefly, reverse transcription was performed with 2 μg poly(A)^+ ^RNA from an individual patient sample in the presence of a mixture of three 3' anchored primers (5'-TTTGCATGCTCGAG-(T)_25_-A/G/C-3') at 42°C for 2 hours. Second strand cDNA was then synthesized with the addition of *E. coli *DNA polymerase I (250 μU/μl; Invitrogen), *E. coli *RNase H (8.5 μU/μl; Invitrogen), and *E. coli *DNA ligase (30 μU/μl; Invitrogen) at 16°C for an additional 2 hours. The double-stranded cDNA libraries were then pooled into healthy and PCa groups. The pooled samples were subjected to *Rsa *I digestion. To identify mRNA species expressed only in patients with PCa, the *Rsa *I digested pooled cDNAs derived from PCa were ligated to specially designed adapters A and B (BD Biosciences Clontech) in two different reactions [25].

To form heterohybrids between two sample populations, the adapter A and adaptor B ligated cDNAs (20 ng) were combined with excess *Rsa *I digested cDNAs (400 ng) from healthy men in two separate reactions, heat-denatured, and hybridized at 68°C for 10 hours. In a second hybridization step, the two separate samples from adapters A and B containing reactions were combined. A fresh aliquot of 150 ng heat-denatured *Rsa *I digested cDNAs derived from healthy men was added to the combined reaction. Hybridization was continued for another 10 hours at 68°C. Commonly expressed sequences between controls and PCa patients formed hybrids in these two sequential hybridization steps. The heterohybrids are less likely to be amplified in the following PCR step due to the design of SSH adaptors [25].

Genes specifically expressed in PCa patients' circulating cells were amplified by two consecutive rounds of PCR according to the procedures reported by Diatchenko *et al*. [25]. The PCR-amplified products were then ligated to the pCRII vector (Invitrogen) followed by transformation. The bacteria were plated on agar plates containing ampicillin and overlaid with X-gal and IPTG. After overnight incubation, white colonies were picked and used for subsequent sequencing reaction. Sequencing results were used to design PCR primer sets to determine the genes' expression levels in healthy controls and PCa patients.

To detect sequences present in circulating cells of healthy men but absent in circulating cells of PCa patients, the initial adaptors ligation reaction was reversed. Aliquots of *Rsa *I digested pooled cDNAs derived from healthy men were ligated to adapters A or B followed by hybridization and PCR amplification as described above.

### RT-PCR confirmation

First strand cDNAs were reverse transcribed from 2.5 μg of the total RNA in the presence of oligo d(T) primer (Invitrogen), 20 μM each of dNTPs, and 200 units of M-MLV reverse transcriptase (Invitrogen). This was done in a total of 50 μl at 42°C for 2 hours. PCR reactions were performed by mixing 1 μl of first-strand cDNAs, 0.2 μM gene-specific 5' and 3' primers (Table 2), and 5 units *Taq *DNA polymerase (Invitrogen) in a total of 50 μl. Reactions were performed by heat activation at 94°C for 2 min, followed by cycling through 94°C for 30 sec, 50–55°C for 1 min, and 72°C for 1 min. The minimal numbers of PCR cycles required for detecting these gene products were first determined and is indicated in Table 2. β-actin (NM_001101) was also amplified and used as an internal control for comparing relative levels of target gene expression. We also included RNA samples without reverse transcription for β-actin amplification to determine levels of genomic DNA contamination. Following gel electrophoresis, images were captured using a Bio-Rad Gel Doc system; and band intensities were analyzed by the Quantity One^® ^software (Bio-Rad).

### Statistical Analysis

Levels of target gene expression were expressed as mean ± standard deviation (SD) following normalization to β-actin. A Student's *t *test was used to compare means of these genes expressions between the healthy controls and PCa patients. A probability value of *p *< 0.05 was considered significant.

## List of abbreviations

CTC, circulating tumor cell

SSH, suppression suppressive hybridization

PCa, prostate cancer.

## Authors' contributions

XL and HKL conducted sample preparations, subtracted library construction, transcript abundance analysis, and data analysis. CW, RM, GS, AM, JK, and CSM participated in patient selection, patient enrollment, and sample collection. DJC, BPK, and HKL participated in study design, data interpretation, and manuscript preparation.
